# Crystal structure of 7-hy­droxy-8-[(4-methyl­piperazin-1-yl)meth­yl]-2*H*-chromen-2-one

**DOI:** 10.1107/S2056989016017217

**Published:** 2016-11-04

**Authors:** Koji Kubono, Ryuma Kise, Yukiyasu Kashiwagi, Keita Tani, Kunihiko Yokoi

**Affiliations:** a Division of Natural Sciences, Osaka Kyoiku University, Kashiwara, Osaka 582-8582, Japan; b Osaka Municipal Technical Research Institute, Osaka 536-8553, Japan

**Keywords:** crystal structure, coumarin, piperazine, hydrogen bonding, π–π inter­actions

## Abstract

In the title compound, C_15_H_18_N_2_O_3_, the coumarin ring is essentially planar, with an r.m.s. deviation of 0.012 Å. An intra­molecular O—H⋯N hydrogen bond forms an *S*(6) ring motif. The piperazine ring adopts a chair conformation. In the crystal, a C—H⋯O hydrogen bond generates a *C*(4) chain motif running along the *c* axis. The chain structure is stabilized by a C—H⋯π inter­action. The chains are linked by π–π inter­actions [centroid–centroid distance of 3.5745 (11) Å], forming a sheet structure parallel to the *bc* plane.

## Abstract 2

There is an intra­molecular O—H⋯N hydrogen bond forming an *S*(6) ring motif in the title compound. In the crystal, mol­ecules are linked by C—H⋯O hydrogen bonds with a *C*(4) chain motif, and also by C—H⋯π inter­actions. The chains are linked by π–π inter­actions, forming a sheet parallel to the *bc* plane.

## Chemical context   

Coumarin (2*H*-chromen-2-one) derivatives have wide applications in diverse areas such as pharmaceuticals (Neyts *et al.*, 2009 ▸), dyes (Hara *et al.*, 2003 ▸) and liquid crystal (Schadt *et al.*, 1996 ▸). Since piperazine is a heterocyclic and aliphatic di­amine, having a flexible structure and a high solubility not only in organic solvents but also in water, its derivatives form complexes with various metal ions in chair and boat conformations. For example, the piperazine ring in a dinuclear zinc(II) complex with a piperazine-based Schiff base adopts a chair form, whereas that in a mononuclear cobalt(III) complex with the same ligand is in a boat form (Cretu *et al.*, 2015 ▸). Moreover, the piperazine ring has recently been utilized as a proton-recognition site in pH-sensitive fluorescent probes (Lee *et al.*, 2014 ▸) and a linker bridging two chromophores in fluorescent ion-sensors (Srivastava *et al.*, 2014 ▸; Jiang *et al.*, 2011 ▸). We are attempting to develop water-soluble chemosensors based on coumarin, and report here the mol­ecular and crystal structure of the title compound.
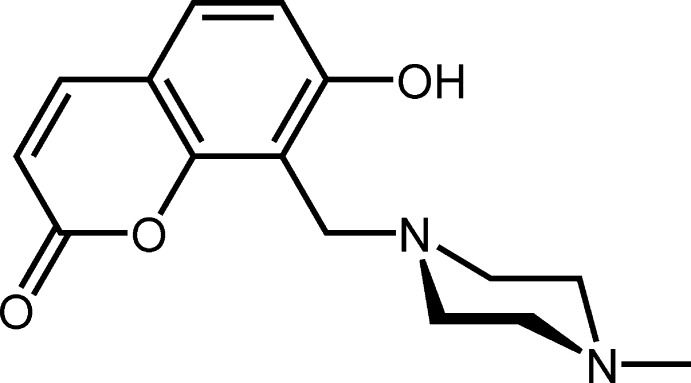


## Structural commentary   

**Figure 1:**
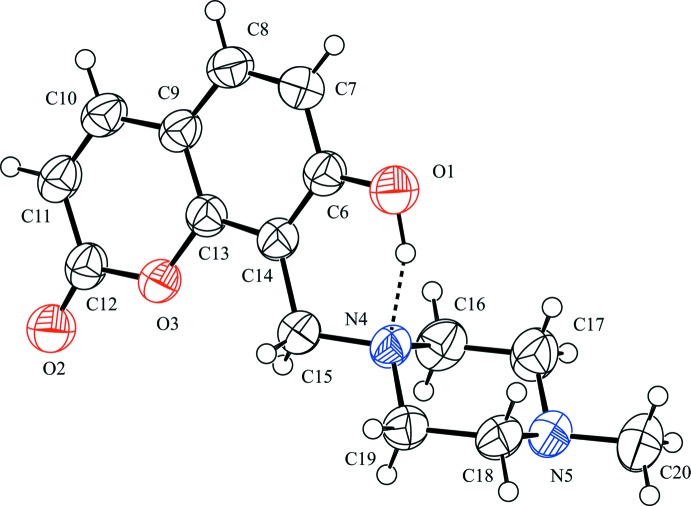
The mol­ecular structure of the title compound, showing the atom labelling. Displacement ellipsoids are drawn at the 50% probability level. The intra­molecular O—H⋯N hydrogen bond is shown as a dashed line.

The mol­ecular structure of the title compound is shown in Fig. 1 ▸. The coumarin ring is almost planar with a maximum deviation of 0.023 (2) Å for atom C6. There is an intra­molecular O—H⋯N hydrogen bond involving the hy­droxy group (O1—H1) and a piperazine N atom (N4), generating an *S*(6) ring motif (Fig. 1 ▸ and Table 1 ▸). The piperazine ring adopts a chair conformation with puckering parameters: *Q* = 0.582 (2) Å, θ = 1.9 (2)° and φ = 22 (7)°. The C16—N4—C15—C14 and C19—N4—C15—C14 torsion angles are −78.8 (2) and 158.52 (16)°, respectively. The bond lengths and angles of the title compound are normal and agree with those values in other Mannich bases of 7-hy­droxy­coumarin (Leong & Vittal, 2010 ▸; Kobayashi *et al.*, 2014 ▸).

## Supra­molecular features   

**Figure 2:**
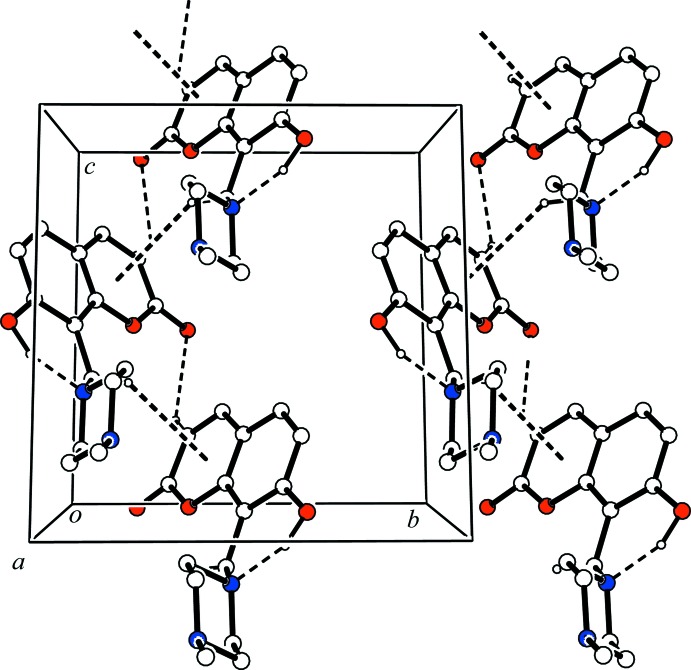
A view along the *a* axis of the crystal packing of the title compound. The hydrogen bonds and C—H⋯π inter­actions are shown as dashed lines. H atoms not involved in these inter­actions have been omitted for clarity.

In the crystal, mol­ecules are linked by a C—H⋯O hydrogen bond (C11—H11⋯O2^i^; symmetry code in Table 1 ▸), forming a *C*(4) chain motif running parallel to the *c* axis. A C—H⋯π inter­action (C15—H15*B*⋯*Cg*1^ii^; *Cg*1 is the centroid of the O3/C9–C13 ring; symmetry code in Table 1 ▸) is also observed in the chain (Fig. 2 ▸). The chains are linked through slipped parallel π–π inter­actions [*Cg*1⋯*Cg*1^iii^ = 3.5745 (11) Å, inter-planar distance = 3.404 Å and slippage = 1.090 Å; symmetry code: (iii) −*x*, −*y*, −*z* + 1], forming a supra­molecular sheet parallel to the *bc* plane.

## Database survey   

A search of the Cambridge Structural Database (CSD, Version 5.37; Groom *et al.*, 2016 ▸) gave 1700 and 85 structures containing coumarin and 7-hy­droxy­coumarin, respectively. Of these structures, the compounds that resemble the title compound are *N*-(7-hy­droxy-4-methyl-8-coumarin­yl)-l-alanine (Leong & Vittal, 2010 ▸) and 8-{[bis­(pyridin-2-ylmeth­yl)amino]­meth­yl}-7-hy­droxy-2*H*-chromen-2-one (Kobayashi *et al.*, 2014 ▸). A search for the fragment methyl­piperazine gave 666 hits, but none contained coumarin.

## Synthesis and crystallization   

The title compound was prepared by modification of the reported procedure (Mazzei *et al.*, 2008 ▸). 1-Methyl­piperazine (0.64 g, 6.4 mmol) and formaldehyde (37% aqueous solution 0.64 mL, 0.64 mmol) in 50 ml of aceto­nitrile was stirred for 30 min at 333 K. To the product obtained was added 7-hy­droxy­coumarin (1.04 g, 0.64 mmol), and the mixture was heated for 3 h at 338 K. After the completion of the reaction, as indicated by TLC, the solvent was removed under vacuum. The residue was suspended in water and extracted with chloro­form, and the extract was washed with a saturated sodium chloride aqueous solution. The organic phase was separated, dried with anhydrous sodium sulfate, and the solvent was removed under vacuum to yield a yellow product. The product was recrystallized from aceto­nitrile solution to obtained colorless crystals of the title compound (yield: 76%). MS (*m*/*z*): [*M* + H]^+^, 275.1. Analysis calculated for C_15_H_18_N_2_O_3_: C 65.68, H 6.61, N 10.21%; found: C 65.40, H 6.45, N 10.06%.

## Refinement   

Crystal data, data collection and structure refinement details are summarized in Table 2 ▸. The hy­droxy H atom was located in a difference Fourier map and freely refined. The C-bound H atoms were positioned geometrically and refined using a riding model: C—H = 0.93–0.97 Å with *U*_iso_(H) = 1.2*U*_eq_(C).

## Supplementary Material

Crystal structure: contains datablock(s) global, I. DOI: 10.1107/S2056989016017217/is5461sup1.cif

Structure factors: contains datablock(s) I. DOI: 10.1107/S2056989016017217/is5461Isup2.hkl

Supporting information file. DOI: 10.1107/S2056989016017217/is5461Isup3.cml

CCDC reference: 1511659

Additional supporting information: crystallographic information; 3D view; checkCIF report

## Figures and Tables

**Table 1:** Hydrogen-bond geometry (Å, °) *Cg*1 is the centroid of the O3/C9–C13 ring.

*D*—H⋯*A*	*D*—H	H⋯*A*	*D*⋯*A*	*D*—H⋯*A*
O1—H1⋯N4	1.02 (3)	1.66 (3)	2.607 (2)	153 (3)
C11—H11⋯O2^i^	0.93	2.59	3.239 (2)	128
C15—H15*B*⋯*Cg*1^ii^	0.97	2.99	3.802 (2)	142

Symmetry codes: (i) 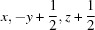; (ii) 
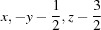
.

**Table 2:** Experimental details

Crystal data	
Chemical formula	C_15_H_18_N_2_O_3_
*M*_r_	274.31
Crystal system, space group	Monoclinic, *P*2_1_/*c*
Temperature (K)	296
*a*, *b*, *c* (Å)	15.3519 (6), 9.4005 (4), 9.9702 (4)
β (°)	106.954 (1)
*V* (Å^3^)	1376.32 (10)
*Z*	4
Radiation type	Mo *K*α
μ (mm^−1^)	0.09
Crystal size (mm)	0.20 × 0.10 × 0.10

Data collection	
Diffractometer	Rigaku R-AXIS RAPID
Absorption correction	Multi-scan (*ABSCOR*; Higashi, 1995 ▸)
*T*_min_, *T*_max_	0.823, 0.991
No. of measured, independent and observed [*F* ^2^ > 2.0σ(*F* ^2^)] reflections	13237, 3136, 1566
*R*_int_	0.037
(sin θ/λ)_max_ (Å^−1^)	0.648

Refinement	
*R*[*F* ^2^ > 2σ(*F* ^2^)], *wR*(*F* ^2^), *S*	0.042, 0.162, 1.05
No. of reflections	3136
No. of parameters	186
H-atom treatment	H atoms treated by a mixture of independent and constrained refinement
Δρ_max_, Δρ_min_ (e Å^−3^)	0.19, −0.18

Computer programs: *RAPID-AUTO* (Rigaku, 2006 ▸), *SIR92* (Altomare *et al.*, 1993 ▸), *SHELXL97* (Sheldrick, 2008 ▸), *PLATON* (Spek, 2015 ▸), *CrystalStructure* (Rigaku, 2016 ▸).

## supplementary crystallographic information

### Crystal data

**Table:**

C_15_H_18_N_2_O_3_	*F*(000) = 584.00
*M**_r_* = 274.31	*D*_x_ = 1.324 Mg m^−^^3^
Monoclinic, *P*2_1_/*c*	Mo *K*α radiation, λ = 0.71075 Å
*a* = 15.3519 (6) Å	Cell parameters from 6828 reflections
*b* = 9.4005 (4) Å	θ = 3.0–27.4°
*c* = 9.9702 (4) Å	µ = 0.09 mm^−^^1^
β = 106.954 (1)°	*T* = 296 K
*V* = 1376.32 (10) Å^3^	Block, colorless
*Z* = 4	0.20 × 0.10 × 0.10 mm

### Data collection

**Table:**

Rigaku R-AXIS RAPID diffractometer	1566 reflections with *F*^2^ > 2.0σ(*F*^2^)
Detector resolution: 10.000 pixels mm^-1^	*R*_int_ = 0.037
ω scans	θ_max_ = 27.4°, θ_min_ = 3.0°
Absorption correction: multi-scan (ABSCOR; Higashi, 1995)	*h* = −19→19
*T*_min_ = 0.823, *T*_max_ = 0.991	*k* = −12→12
13237 measured reflections	*l* = −12→11
3136 independent reflections	

### Refinement

**Table:**

Refinement on *F*^2^	Secondary atom site location: difference Fourier map
*R*[*F*^2^ > 2σ(*F*^2^)] = 0.042	Hydrogen site location: inferred from neighbouring sites
*wR*(*F*^2^) = 0.162	H atoms treated by a mixture of independent and constrained refinement
*S* = 1.05	*w* = 1/[σ^2^(*F*_o_^2^) + (0.0829*P*)^2^ + 0.0112*P*] where *P* = (*F*_o_^2^ + 2*F*_c_^2^)/3
3136 reflections	(Δ/σ)_max_ < 0.001
186 parameters	Δρ_max_ = 0.19 e Å^−^^3^
0 restraints	Δρ_min_ = −0.18 e Å^−^^3^
Primary atom site location: structure-invariant direct methods	

### Special details

**Table:**

Geometry. All esds (except the esd in the dihedral angle between two l.s. planes) are estimated using the full covariance matrix. The cell esds are taken into account individually in the estimation of esds in distances, angles and torsion angles; correlations between esds in cell parameters are only used when they are defined by crystal symmetry. An approximate (isotropic) treatment of cell esds is used for estimating esds involving l.s. planes.
Refinement. Refinement was performed using all reflections. The weighted R-factor (wR) and goodness of fit (S) are based on F^2^. R-factor (gt) are based on F. The threshold expression of F^2^ > 2.0 sigma(F^2^) is used only for calculating R-factor (gt).

### Fractional atomic coordinates and isotropic or equivalent isotropic displacement parameters (Å^2^)

**Table:**

	*x*	*y*	*z*	*U*_iso_*/*U*_eq_	
O1	0.30930 (10)	−0.15085 (16)	0.50405 (16)	0.0643 (4)	
O2	−0.04527 (10)	0.32032 (16)	0.48480 (15)	0.0652 (5)	
O3	0.06794 (9)	0.17001 (13)	0.49705 (13)	0.0516 (4)	
N4	0.26991 (10)	0.05030 (17)	0.31592 (15)	0.0490 (4)	
N5	0.41058 (11)	0.13494 (18)	0.19673 (17)	0.0570 (5)	
C6	0.24973 (13)	−0.0902 (2)	0.5650 (2)	0.0506 (5)	
C7	0.25092 (14)	−0.1386 (2)	0.6984 (2)	0.0552 (5)	
H7	0.2931	−0.2072	0.7432	0.066*	
C8	0.19055 (13)	−0.0858 (2)	0.7631 (2)	0.0535 (5)	
H8	0.1917	−0.1194	0.8513	0.064*	
C9	0.12716 (12)	0.0182 (2)	0.69801 (18)	0.0471 (5)	
C10	0.06032 (14)	0.0772 (2)	0.7567 (2)	0.0525 (5)	
H10	0.0575	0.0456	0.8438	0.063*	
C11	0.00197 (14)	0.1765 (2)	0.6887 (2)	0.0530 (5)	
H11	−0.0407	0.2128	0.7294	0.064*	
C12	0.00365 (13)	0.2289 (2)	0.5539 (2)	0.0507 (5)	
C13	0.12898 (12)	0.0672 (2)	0.56609 (19)	0.0455 (5)	
C14	0.18880 (12)	0.0155 (2)	0.49655 (18)	0.0484 (5)	
C15	0.18271 (13)	0.0648 (2)	0.3490 (2)	0.0588 (6)	
H15A	0.1365	0.0094	0.2822	0.071*	
H15B	0.1639	0.1637	0.3387	0.071*	
C16	0.33329 (15)	0.1654 (2)	0.3767 (2)	0.0638 (6)	
H16A	0.3430	0.1690	0.4772	0.077*	
H16B	0.3077	0.2557	0.3370	0.077*	
C17	0.42259 (15)	0.1407 (3)	0.3465 (2)	0.0698 (7)	
H17A	0.4645	0.2169	0.3876	0.084*	
H17B	0.4490	0.0520	0.3893	0.084*	
C18	0.34652 (13)	0.0235 (2)	0.1345 (2)	0.0565 (5)	
H18A	0.3716	−0.0678	0.1719	0.068*	
H18B	0.3370	0.0222	0.0340	0.068*	
C19	0.25631 (13)	0.0457 (2)	0.16373 (19)	0.0558 (5)	
H19A	0.2291	0.1341	0.1214	0.067*	
H19B	0.2152	−0.0314	0.1227	0.067*	
C20	0.49746 (16)	0.1123 (3)	0.1694 (3)	0.0822 (8)	
H20A	0.5379	0.1894	0.2085	0.099*	
H20B	0.4883	0.1082	0.0700	0.099*	
H20C	0.5236	0.0245	0.2115	0.099*	
H1	0.3043 (18)	−0.092 (3)	0.417 (3)	0.107 (9)*	

### Atomic displacement parameters (Å^2^)

**Table:**

	*U*^11^	*U*^22^	*U*^33^	*U*^12^	*U*^13^	*U*^23^
O1	0.0681 (10)	0.0675 (10)	0.0633 (10)	0.0177 (7)	0.0287 (8)	0.0060 (7)
O2	0.0673 (10)	0.0717 (10)	0.0621 (10)	0.0164 (8)	0.0276 (8)	0.0063 (8)
O3	0.0511 (8)	0.0612 (9)	0.0469 (8)	0.0076 (6)	0.0210 (6)	0.0021 (6)
N4	0.0470 (9)	0.0617 (10)	0.0418 (9)	−0.0007 (8)	0.0184 (7)	−0.0019 (7)
N5	0.0529 (10)	0.0658 (11)	0.0559 (10)	−0.0037 (8)	0.0217 (8)	0.0024 (8)
C6	0.0502 (11)	0.0537 (12)	0.0499 (11)	−0.0005 (9)	0.0179 (9)	−0.0016 (9)
C7	0.0526 (11)	0.0600 (13)	0.0510 (12)	0.0015 (10)	0.0119 (10)	0.0065 (10)
C8	0.0566 (12)	0.0598 (12)	0.0434 (10)	−0.0070 (10)	0.0137 (9)	0.0040 (9)
C9	0.0488 (10)	0.0532 (11)	0.0408 (10)	−0.0086 (9)	0.0155 (9)	−0.0037 (8)
C10	0.0577 (12)	0.0604 (12)	0.0420 (10)	−0.0081 (10)	0.0186 (9)	−0.0039 (9)
C11	0.0557 (12)	0.0601 (13)	0.0489 (11)	−0.0047 (10)	0.0242 (10)	−0.0075 (10)
C12	0.0495 (11)	0.0562 (12)	0.0496 (11)	−0.0030 (10)	0.0197 (10)	−0.0065 (9)
C13	0.0437 (10)	0.0484 (11)	0.0441 (11)	−0.0007 (8)	0.0126 (9)	−0.0013 (8)
C14	0.0460 (10)	0.0572 (12)	0.0437 (10)	0.0004 (9)	0.0159 (9)	−0.0001 (9)
C15	0.0518 (12)	0.0777 (15)	0.0508 (12)	0.0094 (10)	0.0210 (10)	0.0095 (10)
C16	0.0708 (14)	0.0702 (15)	0.0533 (12)	−0.0131 (11)	0.0226 (11)	−0.0149 (10)
C17	0.0592 (13)	0.0911 (18)	0.0593 (14)	−0.0175 (12)	0.0176 (11)	−0.0095 (12)
C18	0.0616 (12)	0.0662 (13)	0.0461 (11)	0.0004 (11)	0.0227 (10)	−0.0011 (9)
C19	0.0546 (12)	0.0709 (14)	0.0426 (11)	−0.0024 (10)	0.0153 (9)	−0.0028 (9)
C20	0.0641 (15)	0.105 (2)	0.0864 (18)	−0.0046 (14)	0.0366 (14)	0.0032 (15)

### Geometric parameters (Å, º)

**Table:**

O1—C6	1.361 (2)	C10—H10	0.9300
O1—H1	1.02 (3)	C11—C12	1.438 (3)
O2—C12	1.215 (2)	C11—H11	0.9300
O3—C13	1.382 (2)	C13—C14	1.390 (2)
O3—C12	1.389 (2)	C14—C15	1.519 (2)
N4—C16	1.463 (2)	C15—H15A	0.9700
N4—C19	1.471 (2)	C15—H15B	0.9700
N4—C15	1.475 (2)	C16—C17	1.505 (3)
N5—C18	1.447 (2)	C16—H16A	0.9700
N5—C17	1.451 (3)	C16—H16B	0.9700
N5—C20	1.453 (3)	C17—H17A	0.9700
C6—C14	1.399 (3)	C17—H17B	0.9700
C6—C7	1.401 (3)	C18—C19	1.511 (2)
C7—C8	1.368 (3)	C18—H18A	0.9700
C7—H7	0.9300	C18—H18B	0.9700
C8—C9	1.399 (3)	C19—H19A	0.9700
C8—H8	0.9300	C19—H19B	0.9700
C9—C13	1.401 (2)	C20—H20A	0.9600
C9—C10	1.433 (3)	C20—H20B	0.9600
C10—C11	1.334 (3)	C20—H20C	0.9600
			
C6—O1—H1	105.1 (15)	N4—C15—H15A	109.1
C13—O3—C12	122.33 (15)	C14—C15—H15A	109.1
C16—N4—C19	109.05 (15)	N4—C15—H15B	109.1
C16—N4—C15	112.12 (15)	C14—C15—H15B	109.1
C19—N4—C15	111.59 (15)	H15A—C15—H15B	107.8
C18—N5—C17	109.58 (15)	N4—C16—C17	109.76 (16)
C18—N5—C20	111.11 (17)	N4—C16—H16A	109.7
C17—N5—C20	110.52 (18)	C17—C16—H16A	109.7
O1—C6—C14	121.37 (17)	N4—C16—H16B	109.7
O1—C6—C7	117.59 (18)	C17—C16—H16B	109.7
C14—C6—C7	121.03 (18)	H16A—C16—H16B	108.2
C8—C7—C6	120.47 (19)	N5—C17—C16	111.21 (18)
C8—C7—H7	119.8	N5—C17—H17A	109.4
C6—C7—H7	119.8	C16—C17—H17A	109.4
C7—C8—C9	120.69 (18)	N5—C17—H17B	109.4
C7—C8—H8	119.7	C16—C17—H17B	109.4
C9—C8—H8	119.7	H17A—C17—H17B	108.0
C8—C9—C13	117.62 (17)	N5—C18—C19	111.37 (16)
C8—C9—C10	124.42 (17)	N5—C18—H18A	109.4
C13—C9—C10	117.96 (18)	C19—C18—H18A	109.4
C11—C10—C9	121.20 (18)	N5—C18—H18B	109.4
C11—C10—H10	119.4	C19—C18—H18B	109.4
C9—C10—H10	119.4	H18A—C18—H18B	108.0
C10—C11—C12	121.47 (18)	N4—C19—C18	109.93 (15)
C10—C11—H11	119.3	N4—C19—H19A	109.7
C12—C11—H11	119.3	C18—C19—H19A	109.7
O2—C12—O3	116.46 (17)	N4—C19—H19B	109.7
O2—C12—C11	126.57 (18)	C18—C19—H19B	109.7
O3—C12—C11	116.97 (18)	H19A—C19—H19B	108.2
O3—C13—C14	116.53 (16)	N5—C20—H20A	109.5
O3—C13—C9	120.07 (16)	N5—C20—H20B	109.5
C14—C13—C9	123.38 (18)	H20A—C20—H20B	109.5
C13—C14—C6	116.77 (16)	N5—C20—H20C	109.5
C13—C14—C15	120.91 (17)	H20A—C20—H20C	109.5
C6—C14—C15	122.18 (16)	H20B—C20—H20C	109.5
N4—C15—C14	112.61 (16)		
			
O1—C6—C7—C8	−177.60 (18)	O3—C13—C14—C15	−3.3 (3)
C14—C6—C7—C8	1.8 (3)	C9—C13—C14—C15	175.31 (17)
C6—C7—C8—C9	−0.6 (3)	O1—C6—C14—C13	178.06 (17)
C7—C8—C9—C13	−1.0 (3)	C7—C6—C14—C13	−1.3 (3)
C7—C8—C9—C10	178.61 (19)	O1—C6—C14—C15	2.5 (3)
C8—C9—C10—C11	179.86 (19)	C7—C6—C14—C15	−176.87 (18)
C13—C9—C10—C11	−0.6 (3)	C16—N4—C15—C14	−78.8 (2)
C9—C10—C11—C12	−0.1 (3)	C19—N4—C15—C14	158.52 (16)
C13—O3—C12—O2	178.67 (16)	C13—C14—C15—N4	155.52 (18)
C13—O3—C12—C11	−0.9 (3)	C6—C14—C15—N4	−29.1 (3)
C10—C11—C12—O2	−178.7 (2)	C19—N4—C16—C17	−59.0 (2)
C10—C11—C12—O3	0.8 (3)	C15—N4—C16—C17	176.87 (17)
C12—O3—C13—C14	179.00 (16)	C18—N5—C17—C16	−57.7 (2)
C12—O3—C13—C9	0.3 (3)	C20—N5—C17—C16	179.51 (18)
C8—C9—C13—O3	−179.94 (16)	N4—C16—C17—N5	59.5 (2)
C10—C9—C13—O3	0.4 (3)	C17—N5—C18—C19	57.0 (2)
C8—C9—C13—C14	1.5 (3)	C20—N5—C18—C19	179.46 (18)
C10—C9—C13—C14	−178.15 (17)	C16—N4—C19—C18	58.4 (2)
O3—C13—C14—C6	−178.98 (16)	C15—N4—C19—C18	−177.23 (16)
C9—C13—C14—C6	−0.3 (3)	N5—C18—C19—N4	−58.1 (2)

### Hydrogen-bond geometry (Å, º)

*Cg*1 is the centroid of the O3/C9–C13 ring.

**Table:**

*D*—H···*A*	*D*—H	H···*A*	*D*···*A*	*D*—H···*A*
O1—H1···N4	1.02 (3)	1.66 (3)	2.607 (2)	153 (3)
C11—H11···O2^i^	0.93	2.59	3.239 (2)	128
C15—H15*B*···*Cg*1^ii^	0.97	2.99	3.802 (2)	142

Symmetry codes: (i) *x*, −*y*+1/2, *z*+1/2; (ii) *x*, −*y*−1/2, *z*−3/2.
